# Under the Same Sky: Connecting Students and Cultures through Circumpolar Nursing Education

**DOI:** 10.3390/healthcare6020050

**Published:** 2018-05-21

**Authors:** Bente Norbye, Lorna Butler, Heather Exner-Pirot

**Affiliations:** 1 Department of Health and Care Sciences, Faculty of Health Sciences, UiT The Arctic University of Norway, 9019 Tromsø, Norway; 2 College of Nursing, University of Saskatchewan, Saskatoon, SK S7N 2Z4, Canada; lorna.butler@usask.ca (L.B.); heather.exnerpirot@usask.ca (H.E.-P.)

**Keywords:** nursing education, Indigenous health, cultural sensitivity, rural and remote regions, circumpolar north

## Abstract

The recruitment and retention of health professionals in rural, remote, and northern regions is an ongoing challenge. The Northern Nursing Education Network brought together nursing students working in rural and remote regions of the circumpolar north in Innovative Learning Institute on Circumpolar Health (ILICH) events to create opportunities for shared learning and expose both students and faculty to local and traditional knowledge that informs health behaviors specific to regions with Indigenous populations. Using participant experience data extracted from program discussions, evaluations, and reflective notes conducted after ILICH events held in 2015–2017, this paper explores how these two-week institutes can contribute to knowledge that is locally relevant yet transferable to rural areas across the circumpolar north. The findings clustered around experiences related to (1) Language as a barrier and an enabler; (2) shared values and traditions across borders; (3) differences and similarities in nursing practice; (4) new perspectives in nursing; and (5) building sustainable partnerships. Students learned more about their own culture as well as others by exploring the importance of language, cultures, and health inequity on different continents. Shared values and traditional knowledge impacted student perspectives of social determinants of health that are highly relevant for nurses working in the circumpolar north.

## 1. Introduction

The provision of health services is impacted by rurality and geographical isolation across the global north [1,2,3,4,5]. An additional challenge that applies to northern regions and worldwide is that only 38% of the nursing workforce practice in rural areas despite such regions being home to almost half of the world’s population [4]. While universities are well positioned to lead the process of developing a local health professional workforce in northern communities, providing on-site opportunities for post-secondary education is a significant challenge due to geographical barriers and a lack of available resources [6,7]. One of the major challenges is that students must often travel far away from their family and communities to receive their nursing education, which not only impacts recruitment, but also retention [2]. Countries such as Norway have been successful in the recruitment and retention of rural nurses by implementing a decentralized nursing education program [8]. Similar successes have been achieved in Canada with a distributed model entitled ‘Learn Where You Live’ [6], based at the University of Saskatchewan (USask). Both programs take nursing education to the students, in smaller rural communities, rather than requiring the students to leave home to attend university.

In distributing a nursing education program situated in a southern or urban region to a northern, rural region, the impact of both culture and context for learners and for responsive pedagogy cannot be overestimated. There has been limited literature to help guide the establishment of a new delivery model using distributed methodologies for hard to reach regions of the world [9,10,11]. However, new networks are established for the improvement of nursing education and rural nursing. The International Council of Nurses (ICN) has developed an International Nursing Education Network to address relevant issues worldwide and a Rural and Remote Nursing Network as a global resource for nurses working in rural areas, see http://www.icn.ch/networks/icn-networks/ [12]. Collaboration between The Arctic University of Norway (UiT) and USask led to the idea of establishing a network in support of northern nursing education. The Northern Nursing Education Network (NNEN) was subsequently established in 2014, involving institutions from seven countries, spanning three continents, and including 13 different post-secondary northern nursing education programs [9]. The aims of the NNEN are to improve the pedagogy of northern nursing education, share best practices, and form a community of students and educators in an annual Innovative Learning Institute on Circumpolar Health (ILICH) to jointly examine and improve the clinical practice of nursing in a northern context [1]. These Institutes, as the key deliverable of the NNEN, were designed to bring together northern nursing students working in rural and remote regions of the north, create opportunities for shared learning, and expose both students and faculty to local and traditional knowledge that informs health behaviors specific to regions with populations of Indigenous peoples. Bringing international nursing students together with the focus of cultural knowledge and the improvement of Indigenous health is a new innovative practice in nursing education [13]. This involved contributing to culturally safe nursing and sharing best practices to achieve optimal health and well-being for circumpolar regions [14]. The opportunity to be involved in observing how different countries address the impact of social determinants of health within local contexts was unprecedented within the existing curricula of the participating nursing programs. This paper describes how these two-week institutes for northern nursing students from Russia, Canada, Greenland, Iceland, Sweden, Finland, and Norway can contribute to knowledge that is locally relevant yet transferable to rural areas across the circumpolar north.

## 2. Materials and Methods 

A retrospective review [11] was used to highlight the outcomes of the three ILICHs, implemented by the NNEN and held on three different continents in 2015, 2016, 2017 (Table 1). The first ILICH was held in the summer of 2015 in Yakutia, Russia. Hosted by North Eastern Federal University (NEFU) in the city of Yakutsk, a total of six nursing students, including two from USask and four from NEFU, and 13 faculty (including two from USask) participated. The second ILICH was hosted by USask in 2016 in the city of Saskatoon and in various communities in northern Saskatchewan, Canada. A total of 14 students from Greenland, Iceland, Finland, Norway, and Yakutia, plus five Canadian students from northern regions of three different provinces, participated, along with 24 educators, including two faculty members from UiT. The third ILICH was hosted by UiT in 2017 and held in the city of Tromsø, Norway. A total of 11 students participated from five countries, including Greenland, Iceland, Russia (Yakutia), Canada (Saskatchewan, Manitoba, Northwest Territories, and Nunavut), Sweden, and Norway (Sápmi), along with 16 faculty including one from USask. The ILICHs held in 2016 and 2017 included the participation of two Master’s students, one in Nursing and one in Pedagogy, who focused on learning objectives relevant to pedagogy, strategies for access to education, and competencies for working in northern rural areas; both were funded by NNEN scholarships. 

The program during the first ILICH in 2015 focused on the impact of isolation on determinants of health in the far north. This included discussing similar and contrasting practices between countries relative to tertiary care for disease management, cultural influences on health practices such as spirituality, ritual beliefs and nursing practice in isolated regions. Formal lectures were provided by an interprofessional team of faculty from the health sciences at NEFU. Participants were also immersed in the culture of the region through historical and experiential learning by engaging with community members in remote villages and nursing stations and sessions with curators of museums to teach the history of the region including social, political and economic influences. In 2016 and 2017, at University of Saskatchewan and UiT, respectively, the programs were divided into two main parts. The first week had lectures on cultural sensitivity, about the Indigenous population(s) in the host region and specific warming up activities for the students. In addition, specific seminars were held at museums and at Indigenous Heritage cultural places of interest, with contributions from specialists in their fields. Seminars were topics on specific health issues among Indigenous populations and northern regions, such as tuberculosis (TB) treatment, oral health among children, the use of telehealth to connect rural with more urban hospitals with an emphasis on best practices. Sessions provided by community leaders and Indigenous leaders focused on the inclusion of Indigenous ways of knowing in health and health care delivery, emphasizing respect for differences in culture and the local context in which care is to be provided. The seminars were held both by university lecturers from the nursing programs and with specialists in their field and having an Indigenous background. The second weeks were field trips and visits to northern, rural areas, where meeting nurses and people living in the area were central. For those students who participated in 2015 and 2016, experiential learning opportunities involved spending time with Elders, First Nations and Metis Chiefs, community members and children. Students travelled across rough terrain and unpaved roadways to access communities, thus experiencing the challenges of remoteness and access to resources readily available within their respective universities and home communities. Student learning was an important part of the ILICH. The programs emphasized students’ reflective journaling about their observations of working and living in the rural areas. At the completion of the 2016 Institute, students were required to present their assessment of the field experience. In 2017, a similar student-led seminar was held at Riddu, the International Indigenous Festival in Kåfjord in the North of Norway, as UiT is a collaborative partner with the Festival. The students from the ILICH in 2017 were included in the program for an open seminar and presented their knowledge concerning Indigenous peoples’ history. The students attended to the health-related challenges they, as nurses, would attend to, and the importance of this knowledge in making a difference. 

One goal of the ILICHs was to improve and enhance northern nursing opportunities so as to develop a stable northern nursing workforce. Northern and rural health regions face many challenges in attracting and maintaining a stable, culturally competent health workforce [15]. Students participating in the Institutes were enrolled in nursing education programs in northern jurisdictions in the hope that they would remain and join their local nursing workforce. The two Canadian students involved in the 2015 ILICH have both remained in their communities and have full-time positions in nursing practice. Data for participants in the 2016 and 2017 Institutes will be available by the end of 2018. 

The students were recruited to the ILICH among their Northern Nursing Education network members and there were no inclusion criteria regarding whether the students had an Indigenous background or not. Scandinavian regulations prohibit the collection of data about people’s ethnicity due to discrimination against the Sami population. Nevertheless, among the students attending the ILICH, more than 80% self-reported coming from different Indigenous populations across the circumpolar north. 

Data referenced in this review were extracted from comments submitted by the two Canadian student participants of the 2015 ILICH, written summative evaluations from all 14 students student participants of the 2016 ILICH, 10 reflective logs completed by students seven months after the 2017 ILICH, and four face-to-face interviews with students during and after the field trip portion of the 2017 ILICH. Additionally, students had submitted written comments concerning lessons learned from their experiences at the ILICH from all three Institutes, which were included. These data were combined and analyzed using content analysis [16].

Participating universities were encouraged to use the ILICH syllabus as course content that could be used as credit towards the student’s nursing program. To that end, student statements and feedback to the ILICH teams were provided after the program was completed, and therefore could in no way affect the assessment of students’ progress at the Institute. The reflective notes from students after the 2017 field course were collected seven months post completion of the Institute. Although students’ comments were grouped, references to home country in the quotations shared below are included to best reflect the students’ own words and to reflect how they think about cultures that differ from those with which they are familiar. 

## 3. Results

The findings represent the outcomes of the ILICHs as expressed by the students. Comments clustered around experiences related to: (1) communicating beyond a language; (2) shared values and traditions across borders; (3) differences and similarities in nursing practice; (4) new perspectives in nursing; and (5) building sustainable partnerships. A recurring theme was relationship building. Students formed new partnerships that could lend support to their practice as they begin careers as new graduates working in rural, remote, and northern communities. Despite great geographical distances, technology offered real-time opportunities for connecting globally. 

### 3.1. Language as a Barrier and an Enabler 

English was the working language of the ILICHs, but proficiencies varied considerably across participants. Attending an ILICH meant that the student participants had to experience new languages and teach each other how to express their thoughts in both words and gestures that did not reflect their own first language. The inability to express oneself so that others could comprehend, as well as trying to understand each other when diction and phrasing were unclear, presented challenges for all students. 

Many of the nursing students from the Nordic countries were able to speak a second language, with differing levels of fluency. A learning outcome of the ILICHs was the opportunity for students to experience the frustration that many of their patients in their home communities also experience when required to travel to urban and/or southern regions for health care. Further, for nurses working internationally or in a new culture, the need to understand words, gestures, and symbolic representations of health, illness, treatment, and community became clear to the students. When one cannot speak the native language or misunderstands what is being expressed, the ability/inability of the nurse to meet the needs of the patient can be frustrating for both as one students related the language barrier to her home community and the access of health care. Some expressed that it was important to know the languages of the people, as this knowledge was key for trust between the nurse and their patients. Understanding the language was also viewed as providing a cultural link for understanding Indigenous ways of knowing and related health practices. 

### 3.2. Shared Values and Traditions across Borders

Students attending the ILICHs had firsthand knowledge of their own culture and values, and many of them expressed their own sense of Indigenous belonging. Conversations often centered around the values of being part of an extended family and the meaning of the land. There was an inherent appreciation by all students of cultural background, Indigenous and local values, the importance of family, and the meaning of land to future health and prosperity. Students had the opportunity to compare and contrast each of these and the application to their future work as nurses:

I felt the content of the seminars and activities to be relevant towards my nursing practice. I particularly enjoyed learning about how they overcome barriers to access to health care. We all tended to experience similar challenges when it comes to the isolation and remoteness.

(written reflection, undergraduate student 2017)

Another student expressed:

The lack of trust and cultural differences often lead to people from the minority groups not seeking medical help or treatment. Without relationships of trust, it is difficult for health workers to get the information you need to attend to the issues.

(interview, July 2017)

Not all northern countries have an Indigenous population, yet the focus was still relevant, as students could refer to the knowledge in nursing practice. The ILICHs provided the unique opportunity for students to learn not only about diversity, but also about strengths and vulnerabilities of mixed populations, as the participating countries provided tacit knowledge from non-Indigenous (Iceland), Indigenous minority (Norway, Yakutsk, parts of Canada), and Indigenous majority (Greenland, parts of Canada) circumstances:

I was delighted to discover that my Inuvialuit ancestors utilized the same sod style houses as in those traditionally used by the Sami. Also, hearing about the cultural assimilation and intergenerational trauma inflicted was surprisingly similar to the same struggles that Indigenous Peoples of Canada have endured. I was empowered learning about the process towards self-determination that the Sami have, and continue to, accomplish. It gives me hope that with strength and determination my own people are headed in the right direction towards truth and reconciliation.

(written reflections, undergraduate student 2017)

### 3.3. Differences and Similarities in Nursing Practice

When students come to a new continent, they initially see differences, such as language, food, and traditional symbols. Exposure to the health care system in each country resulted in a consistent response about how health care was delivered differently across all countries. However, the students also learned they had more in common than expected. These differences and the similarities were discussed among the students as they explored experiences during the ILICH:

I was amazed to hear that my colleague from Greenland and I shared cultural similarities such as hunting styles, clothing, language, and traditional diet. I shared traditional stories regarding Aurora Borealis with my colleagues from Russia and found that their stories were similar—they had a moral or an important lesson to be passed on. My colleagues from Russia and I also discussed similarities in traditional beading and handiwork when they noticed my beaded purse.

(written reflections, undergraduate student 2017)

Greater differences were discovered among the students with respect to their roles as nurses and in terms of procedures and responsibilities. The differences were greater when the students compared treatment and care across countries. Students learned that the treatment regimes used to care for individuals with tuberculosis were the same, but the method of care delivery was quite different, as one student expressed:

I was really surprised that they still had TB sanatoriums in Yakutsk and they were equally surprised that we did not.

(written reflection, undergraduate student 2015)

This was further contrasted by population, as students learned about the nomadic lifestyle followed by portions of the population as well as the lack of transportation and accessible roadways in northeastern Russia and northern Saskatchewan. 

### 3.4. New Perspectives in Nursing 

Connecting nursing students from different parts of the circumpolar north afforded each student the opportunity to reflect on the values, beliefs, and health practices of their own country. For some, the lessons learned were a revelation, as students tried to express themselves. Participation in an ILICH was more than just traveling to a new country; the expectations to examine their own understanding of nursing and health allowed them to rethink context, culture, and health behaviors in their home communities. The field trip to a local community made the students aware of how limited resources could be used, and made the teaching more relevant for them as future nurses as they saw how relatively limited resources could be utilized. The students learned about the challenges in other’s countries and saw the link to Indigenous people’s history, reflecting on how today’s issues are a product of struggles of the past. The experience of being with an international group expanded students’ body of knowledge with respect to what was important for people across the circumpolar regions, as well as how nursing involves dealing with people who might have lost their language and their cultural values due to colonization across borders:

I met a lot of amazing people whom are studying the same thing as I’m and working with the same issues as I’m but with different resources. In our time together we did a project about how united we are as nurses and that is something that I will always value.

(written reflection, undergraduate student 2017)

Another student expressed:

Cultural awareness is a huge component of nursing. This trip helped me become more aware of my own culture, beliefs, values and perceptions. In nursing, you will care for individuals from many different backgrounds. Therefore, it is essential to become culturally aware so you can respect other cultures.

(written reflection, undergraduate student 2017)

Studying in the far north of Canada and being able to relate to other nursing students at the ILICHs extended participant understanding of what the nurses from these various regions had in common. 

### 3.5. Building Sustainable Partnerships

Students participating in the ILICHs came from different northern communities across three continents, including some rural and some very remote regions. Once they arrived at the ILICH, the students quickly became part of a team required to live and work together as a small community of northern nursing students. During each ILICH, students formed relationships that stretched beyond borders and cultural differences. For some, partnerships emerged that have continued to grow as ongoing collaborations occur within their respective universities and hospitals. Others have formed partnerships at a more global level using social media. The connectivity evolved as they learned to appreciate that their health service and nursing practices were relevant to others. The relationships that formed and the partnerships among the students and organizations involved have contributed to the NNEN motivation to sustain and expand the network to be more inclusive of other health disciplines that comprise a northern health workforce. This community of nursing students, which began through the ILICHs, has continued to expand into a bigger community for future practice:

It was a great way to form partnerships with future nurses from around the world who share a common goal of providing better health care to their Indigenous populations The Sami people have a lot in common with Canadian aboriginal peoples, such as being sent to boarding school and the attempt to assimilate them with the Norwegian culture.

(interview, undergraduate student 2017)

Others expressed that learning together naturally forms a connection that is valuable during their time as students but also beyond as their careers evolve.

Attending the summer institute in Tromsø allowed me to gain insight into other cultures and to embrace my own. I got to compare Norway and Canada’s Indigenous peoples culture and history, and surprisingly found many similarities. Attending the Riddu Riddu Cultural Festival was also a highlight of the trip. There were multicultural groups from all over the world. It was inspiring to see so many people celebrating their roots and identity.

(written reflection, undergraduate student 2017)

Students highlighted the impact of the field trips to local communities on their learning. The ILICHs all had a strong focus on student engagement with rural and remote communities outside the main center where health services and tertiary care hospitals are situated. The goal was to expose students to social determinants of health as experienced by those seeking health care. Examples include long distances to access care, modes of transportation, road conditions, housing, economics, and education. Meeting with community leaders, Elders, knowledge keepers, children, and health professionals provided students with further opportunities to share traditional meals, talk with nurses from the local health services, appreciate local customs, and learn the history and meaning of traditional clothing and accessories. The field trip included nursing students engaging and presenting at Riddu Riđđu Festivàla, the international Indigenous Festival in the coastal Sami Gáivuotna–Kåfjord municipality in northern Norway. The students presented their chosen subject of Indigenous people’s rights for access to health care.

## 4. Discussion

The participants across the three years of the ILICH will likely experience barriers and challenges related to working in smaller communities after graduation. Confronting issues of health care access and working with people with inter-generational struggles contributes to the participants’ preparation and cultural awareness. 

Expressing themselves in a language other than their mother tongue also provided lessons. This began on the first day of each Institute, when students were asked to give a personal introduction about their life, their culture, and why they were attending the ILICH. The mutual language was English, though only the Canadian students had English as their first language. Knowledge of the language became important for the students as they experienced first-hand how it was to struggle to be understood and also how important language is for establishing trust between the nurse and patient. Learning about the languages also included knowledge of cultural values and expressions related to colloquial terms and ways of living in smaller communities. 

Differences and similarities were both noted by students who were visiting a new continent, while at the same time, in-country participants learned new perspectives about their “home” regions from others. Within a two-week time frame, students were able to share common issues and explore new ways of dealing with health issues. Having access to a nursing education program was of great importance to the people living in rural communities. The students also explored how music could connect people by expressing sounds and emotions using traditional instruments and song. For example, two Russian students performed with a traditional Siberian Indigenous instrument called a Khomus, a reed instrument where the sound made by the tongue on the reed imitates horse hooves. Using this approach, the students were able to express where they came from while the audience responded in recognition that they could hear and visualize the horses galloping over the land.

The program review demonstrates the impact of the opportunity to provide experiences for northern and Indigenous students to explore different cultures and thereby compare their own with a different country. The reflective process, explaining the values and important aspects of their own cultures to other students, contributed to understanding their own culture as well as learning about the culture of the host country. This was triggered by discovering the similarities and differences across international borders and even coming together from different continents. As many of the students are likely to work in rural areas after graduation, the feeling of unity among the nurses might help them to understand the struggles of rural communities with people from Indigenous communities. 

Perhaps one of the most significant learning opportunities was to appreciate that, while all communities have challenges, focusing on the strengths of the people and what each community and each country has to offer for shared understanding demonstrates respect for the people and an acknowledgement that each student and each country has much to offer. The ILICHs provided multiple occasions to connect nursing students across the circumpolar north and show them differing perspectives with new ideas and methods to address issues related to social determinants of health. A measure of the relationships that have evolved, the ongoing consultations, and continued contribution to shared learning across the north is the ongoing interactions on social media. Through their experiences at the ILICHs, students discovered that they belong to a northern network relevant for future nurses even if they study and work in a rural area in their home land. 

Nursing students from rural areas are generally more mature students with family commitments and work obligations [8]. Therefore, they have less opportunity to engage in student exchange programs due to the cost of travel; the distances also make travel time-consuming. The nursing students from rural areas are likely to eventually work as nurses in rural areas, and as such will have less support from a network of health professions than those working in more urban centers and hospitals [17]. This study shows that an international contingent of nursing students coming together for a two-week summer field course can make an important contribution to their awareness, as it highlights understanding of their own and other cultures relevant for nursing practice. Mainstream nursing education is less concerned with rural nursing as a specialization and might neglect relatively important aspects of rural nursing. Further, special efforts, both in undergraduate and postgraduate programs, should support rural components of nursing, as recommended by WHO [4], by designing professional development programs that meet the needs of rural health workers. 

With respect to limitations of this work, the authors of this article acknowledge they were involved in establishing the ILICHs, and as such might have a bias with respect to the overall results. However, all responses came from students after the ILICHs were completed and none of the authors met the students as part of their nursing education. Student grades would not be affected by the feedback provided.

## 5. Conclusions

Northern nursing students across seven countries and three continents, representing different backgrounds, ethnicity, nationalities, and languages, participated in three iterations of a two-week intensive learning opportunity on health in the circumpolar north. By exploring differences and similarities between cultures, languages, traditional knowledge, and various programs of nursing education, the experiences made an impact on student perspectives of social determinants of health and well-being relevant to the local context within which health care and health behaviors are practiced in their home countries and communities. These new perspectives add to their understanding of nursing and the contribution they can make to building safe, healthy communities at home and in the global north. The recruitment of students from rural areas benefits the retention of nurses. However, the extended understanding of where they come from and what purpose they, as nurses, serve for a population were highlighted and understood differently as a result of an intensive summer course as the ILICH as each ILICH focused on expertise at the local level, with regional and national relevance within the host country. Pride in sharing traditional knowledge and inter-country exchange of ideas acknowledging the contribution of northern and Indigenous ways of knowing have been foundational to the development of the Institutes. 

## Figures and Tables

**Table 1:** Participation in the Innovative Learning Institute on Circumpolar Health, 2015-2017.

Host University/Year	Countries Represented	Nursing Students Participating	Faculty/Educators Involved in Teaching
North-Eastern Federal University in Yakutsk/2015	2	6	13
University of Saskatchewan/2016	6	14	24
UiT The Arctic University of Norway/2017	6	11	16
